# (*Z*)-Ethyl 3-(4-chloro­benzamido)-2-cyano-3-(4-fluoro­phen­yl)acrylate

**DOI:** 10.1107/S1600536808041469

**Published:** 2008-12-10

**Authors:** Dehua Zhang, Xiaoyan Zhang, Lijuan Guo

**Affiliations:** a Department of Chemistry and Environmental Engineering, Hubei Normal University, Huangshi 435002, People’s Republic of China; b School of Mathematics and Physics, Huangshi Institute of Technology, Huangshi 435003, People’s Republic of China; c Department of Chemistry, Changsha Medical University, Changsha 410219, People’s Republic of China

## Abstract

The title compound, C_19_H_14_ClFN_2_O_3_, was prepared by the reaction of ethyl (*Z*)-3-amino-2-cyano-3-(4-fluoro­phen­yl)acrylate and 4-chloro­benzoyl chloride. The dihedral angle between the chloro­benzene and fluoro­benzene rings is 66.18 (19)°. In addition to an intra­molecular N—H⋯O hydrogen bond, there are inter­molecular C—H⋯O and C—H⋯N hydrogen bonding inter­actions, which stabilize the crystal structure.

## Related literature

For the agrochemical activity of the title compound, see: Heller *et al.* (2004 ▶); Ibers & Hamilton (1964 ▶).
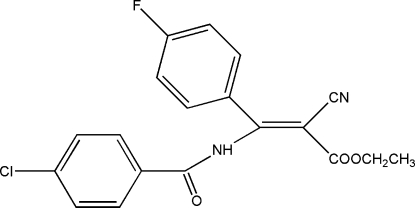


## Experimental

### 

#### Crystal data

C_19_H_14_ClFN_2_O_3_
*M* *_r_* = 372.77
Monoclinic, 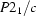

*a* = 6.1429 (5) Å
*b* = 13.1555 (6) Å
*c* = 22.9263 (10) Å
β = 92.280 (4)°
*V* = 1851.27 (19) Å^3^
*Z* = 4
Mo *K*α radiation
μ = 0.24 mm^−1^
*T* = 298 (2) K
0.40 × 0.20 × 0.10 mm

#### Data collection

Bruker SMART 1000 CCD area-detector diffractometer
Absorption correction: none
23332 measured reflections
3259 independent reflections
2138 reflections with *I* > 2σ(*I*)
*R*_int_ = 0.053

#### Refinement

*R*[*F* ^2^ > 2σ(*F* ^2^)] = 0.065
*wR*(*F* ^2^) = 0.140
*S* = 1.07
3259 reflections
236 parameters
H-atom parameters constrained
Δρ_max_ = 0.25 e Å^−3^
Δρ_min_ = −0.23 e Å^−3^

### 

Data collection: *SMART* (Bruker, 1997 ▶); cell refinement: *SAINT* (Bruker, 1999 ▶); data reduction: *SAINT*; program(s) used to solve structure: *SHELXS97* (Sheldrick, 2008 ▶); program(s) used to refine structure: *SHELXL97* (Sheldrick, 2008 ▶); molecular graphics: *SHELXTL* (Sheldrick, 2008 ▶); software used to prepare material for publication: *SHELXTL*.

## Supplementary Material

Crystal structure: contains datablocks I, global. DOI: 10.1107/S1600536808041469/rz2277sup1.cif

Structure factors: contains datablocks I. DOI: 10.1107/S1600536808041469/rz2277Isup2.hkl

Additional supplementary materials:  crystallographic information; 3D view; checkCIF report

## Figures and Tables

**Table 1:** Hydrogen-bond geometry (Å, °)

*D*—H⋯*A*	*D*—H	H⋯*A*	*D*⋯*A*	*D*—H⋯*A*
N1—H1⋯O2	0.86	2.00	2.668 (3)	134
C3—H3⋯N2^i^	0.93	2.53	3.314 (5)	143
C6—H6⋯O1^ii^	0.93	2.41	3.170 (4)	140
C14—H14⋯N2^iii^	0.93	2.55	3.463 (5)	169

Symmetry codes: (i) 
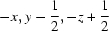
; (ii) 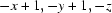; (iii) 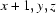.

**Fig. 1.**
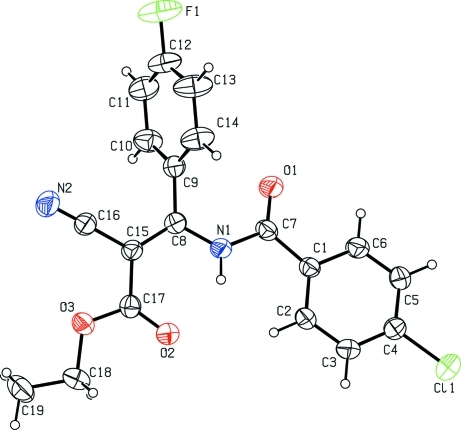
The molecular structure of The title compound, showing 30% probability displacement ellipsoids and the atom-numbering scheme.

**Fig. 2.**
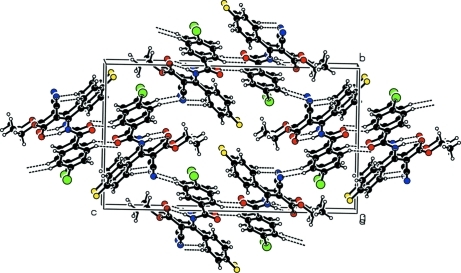
Packing diagram of the title compound, viewed approximately along the a axis. Hydrogen bonds are shown as dashed lines.

## supplementary crystallographic information

### Comment

Recently, 2-cyanoacrylates have been extensively used as agrochemicals because of their unique mechanism of action and good environmental profiles. The title compound is useful as an inhibitor of Pyricularia oryzae, Rhizoctonia solani, Botrytis cinerea and Gibberella zeae (Heller *et al.*, 2004; Ibers & Hamilton, 1964).

In the title compound (Fig.1), all bond lengths and angles are unexcepional. The aromatic rings of the chlorobenzene and fluorobenzene groups form a dihedral angle of 66.18 (19)°. The molecular conformation is stabilized by an intramolecular N—H···O hydrogen bond (Table 1). The crystal packing is governed by C—H···O and C—H···N hydrogen interactions (Fig.2) resulting in a three-dimensional network.

### Experimental

To a solution of ethyl (2*Z*)-3-amino-2-cyano-3-(4-fluorophenyl)acrylate (1.17 g, 0.0050 mol) in CH_2_Cl_2_ (18 ml), 4-chlorobenzoyl chloride (2.63 g, 0.015 mol) was added. Subsequently, Et_3_N (1.52 g, 0.015 mol) was dropped into the solution under stirring. The reaction mixture was then heated to reflux, stirred for 4 h and cooled to room temperature. The reaction solution was filtered off and some white solid was separated. The organic phase was washed with water and then dried over Na_2_SO_4_. After removal of the solvent, a brown dope was obtained. The title compound was isolated by column chromatography using ethyl acetate/light petroleum (1:6 v/v) as eluent. Single crystals suitable for X-ray analysis were obtained by slow evaporation at room temperature of an ethyl acetate/petroleum ether (3:1 v/v) solution after 45 days.

### Refinement

All H atoms were placed at calculated positions and refined using a riding model, with C—H = 0.93-0.97 Å, N—H = 0.86 Å and with *U*_iso_(H) = 1.2 *U*_eq_(C, N) or 1.5 *U*_eq_(C) for methyl H atoms.

### Crystal data

**Table:**

C_19_H_14_ClFN_2_O_3_	*F*(000) = 768
*M**_r_* = 372.77	*D*_x_ = 1.337 Mg m^−^^3^
Monoclinic, *P*2_1_/*c*	Mo *K*α radiation, λ = 0.71073 Å
Hall symbol: -P 2ybc	Cell parameters from 1253 reflections
*a* = 6.1429 (5) Å	θ = 3.1–20.3°
*b* = 13.1555 (6) Å	µ = 0.24 mm^−^^1^
*c* = 22.9263 (10) Å	*T* = 298 K
β = 92.280 (4)°	Block, colourless
*V* = 1851.27 (19) Å^3^	0.40 × 0.20 × 0.10 mm
*Z* = 4	

### Data collection

**Table:**

Bruker SMART 1000 CCD area-detector diffractometer	2138 reflections with *I* > 2σ(*I*)
Radiation source: fine-focus sealed tube	*R*_int_ = 0.053
graphite	θ_max_ = 25.0°, θ_min_ = 1.8°
phi and ω scans	*h* = −7→7
23332 measured reflections	*k* = −15→15
3259 independent reflections	*l* = −24→27

### Refinement

**Table:**

Refinement on *F*^2^	Primary atom site location: structure-invariant direct methods
Least-squares matrix: full	Secondary atom site location: difference Fourier map
*R*[*F*^2^ > 2σ(*F*^2^)] = 0.065	Hydrogen site location: inferred from neighbouring sites
*wR*(*F*^2^) = 0.140	H-atom parameters constrained
*S* = 1.07	*w* = 1/[σ^2^(*F*_o_^2^) + (0.0335*P*)^2^ + 1.3651*P*] where *P* = (*F*_o_^2^ + 2*F*_c_^2^)/3
3259 reflections	(Δ/σ)_max_ = 0.001
236 parameters	Δρ_max_ = 0.25 e Å^−^^3^
0 restraints	Δρ_min_ = −0.23 e Å^−^^3^

### Special details

**Table:**

Geometry. All e.s.d.'s (except the e.s.d. in the dihedral angle between two l.s. planes) are estimated using the full covariance matrix. The cell e.s.d.'s are taken into account individually in the estimation of e.s.d.'s in distances, angles and torsion angles; correlations between e.s.d.'s in cell parameters are only used when they are defined by crystal symmetry. An approximate (isotropic) treatment of cell e.s.d.'s is used for estimating e.s.d.'s involving l.s. planes.
Refinement. Refinement of *F*^2^ against ALL reflections. The weighted *R*-factor *wR* and goodness of fit *S* are based on *F*^2^, conventional *R*-factors *R* are based on *F*, with *F* set to zero for negative *F*^2^. The threshold expression of *F*^2^ > σ(*F*^2^) is used only for calculating *R*-factors(gt) *etc*. and is not relevant to the choice of reflections for refinement. *R*-factors based on *F*^2^ are statistically about twice as large as those based on *F*, and *R*- factors based on ALL data will be even larger.

### Fractional atomic coordinates and isotropic or equivalent isotropic displacement parameters (Å^2^)

**Table:**

	*x*	*y*	*z*	*U*_iso_*/*U*_eq_	
C1	0.4513 (5)	0.4421 (2)	0.11848 (12)	0.0510 (7)	
C2	0.4752 (5)	0.3939 (2)	0.17187 (14)	0.0613 (9)	
H2	0.3761	0.4064	0.2006	0.074*	
C3	0.6452 (6)	0.3273 (3)	0.18272 (14)	0.0670 (9)	
H3	0.6599	0.2942	0.2185	0.080*	
C4	0.7924 (5)	0.3099 (2)	0.14056 (15)	0.0626 (9)	
C5	0.7716 (6)	0.3559 (3)	0.08732 (15)	0.0696 (9)	
H5	0.8718	0.3431	0.0589	0.084*	
C6	0.6006 (6)	0.4213 (2)	0.07632 (14)	0.0673 (9)	
H6	0.5848	0.4523	0.0399	0.081*	
C7	0.2778 (5)	0.5173 (2)	0.10220 (14)	0.0582 (8)	
C8	−0.0191 (5)	0.6171 (2)	0.14507 (13)	0.0531 (8)	
C9	−0.0435 (5)	0.6899 (2)	0.09582 (13)	0.0557 (8)	
C10	−0.2251 (6)	0.6897 (3)	0.05965 (16)	0.0790 (11)	
H10	−0.3308	0.6400	0.0642	0.095*	
C11	−0.2547 (7)	0.7618 (3)	0.01659 (16)	0.0873 (12)	
H11	−0.3779	0.7598	−0.0083	0.105*	
C12	−0.1070 (8)	0.8340 (3)	0.01079 (16)	0.0870 (13)	
C13	0.0819 (9)	0.8367 (3)	0.04453 (19)	0.1072 (16)	
H13	0.1867	0.8863	0.0388	0.129*	
C14	0.1124 (7)	0.7635 (3)	0.08740 (17)	0.0879 (12)	
H14	0.2395	0.7638	0.1108	0.106*	
C15	−0.1571 (5)	0.6200 (2)	0.18996 (13)	0.0539 (8)	
C16	−0.3251 (6)	0.6945 (3)	0.18956 (14)	0.0667 (9)	
C17	−0.1424 (5)	0.5506 (2)	0.24042 (13)	0.0561 (8)	
C18	−0.3118 (6)	0.4975 (3)	0.32660 (15)	0.0742 (10)	
H18A	−0.1840	0.5102	0.3518	0.089*	
H18B	−0.3126	0.4264	0.3155	0.089*	
C19	−0.5130 (7)	0.5229 (4)	0.35748 (17)	0.1065 (15)	
H19A	−0.5114	0.5937	0.3677	0.160*	
H19B	−0.5193	0.4826	0.3923	0.160*	
H19C	−0.6383	0.5088	0.3324	0.160*	
Cl1	1.00991 (17)	0.22922 (8)	0.15591 (5)	0.0969 (4)	
F1	−0.1365 (6)	0.90693 (18)	−0.03083 (11)	0.1356 (11)	
N1	0.1499 (4)	0.54859 (19)	0.14698 (10)	0.0595 (7)	
H1	0.1799	0.5216	0.1805	0.071*	
N2	−0.4597 (6)	0.7536 (3)	0.19023 (14)	0.0981 (12)	
O1	0.2500 (5)	0.5501 (2)	0.05344 (10)	0.0940 (9)	
O2	0.0048 (4)	0.49015 (17)	0.24988 (10)	0.0701 (6)	
O3	−0.3085 (4)	0.56200 (16)	0.27479 (9)	0.0628 (6)	

### Atomic displacement parameters (Å^2^)

**Table:**

	*U*^11^	*U*^22^	*U*^33^	*U*^12^	*U*^13^	*U*^23^
C1	0.0583 (19)	0.0492 (16)	0.0460 (17)	−0.0001 (14)	0.0068 (15)	0.0007 (14)
C2	0.065 (2)	0.064 (2)	0.056 (2)	0.0058 (17)	0.0166 (17)	0.0054 (16)
C3	0.076 (2)	0.070 (2)	0.055 (2)	0.0070 (19)	0.0057 (18)	0.0142 (17)
C4	0.066 (2)	0.0532 (18)	0.069 (2)	0.0047 (16)	0.0076 (18)	0.0025 (16)
C5	0.077 (2)	0.073 (2)	0.061 (2)	0.0152 (19)	0.0198 (18)	0.0022 (18)
C6	0.085 (3)	0.066 (2)	0.0522 (19)	0.0133 (19)	0.0115 (18)	0.0070 (16)
C7	0.069 (2)	0.0603 (19)	0.0460 (19)	0.0017 (16)	0.0124 (16)	0.0019 (15)
C8	0.060 (2)	0.0509 (17)	0.0482 (18)	0.0018 (15)	−0.0006 (15)	−0.0051 (14)
C9	0.064 (2)	0.0523 (18)	0.0514 (18)	0.0036 (16)	0.0030 (16)	−0.0038 (15)
C10	0.083 (3)	0.086 (3)	0.067 (2)	−0.013 (2)	−0.011 (2)	0.013 (2)
C11	0.093 (3)	0.099 (3)	0.067 (2)	0.003 (3)	−0.025 (2)	0.008 (2)
C12	0.137 (4)	0.056 (2)	0.066 (2)	0.008 (2)	−0.021 (3)	0.0120 (19)
C13	0.150 (4)	0.066 (2)	0.102 (3)	−0.032 (3)	−0.038 (3)	0.026 (2)
C14	0.109 (3)	0.069 (2)	0.083 (3)	−0.014 (2)	−0.032 (2)	0.012 (2)
C15	0.060 (2)	0.0543 (17)	0.0471 (18)	0.0084 (15)	0.0011 (15)	−0.0003 (14)
C16	0.073 (2)	0.077 (2)	0.050 (2)	0.019 (2)	0.0033 (17)	0.0008 (17)
C17	0.057 (2)	0.0577 (19)	0.0537 (19)	0.0008 (16)	0.0027 (16)	−0.0077 (16)
C18	0.086 (3)	0.076 (2)	0.060 (2)	−0.013 (2)	0.0093 (19)	0.0138 (18)
C19	0.095 (3)	0.155 (4)	0.072 (3)	0.002 (3)	0.028 (2)	0.021 (3)
Cl1	0.0849 (7)	0.0965 (7)	0.1095 (8)	0.0329 (6)	0.0085 (6)	0.0214 (6)
F1	0.226 (3)	0.0791 (16)	0.0970 (18)	0.0063 (18)	−0.0458 (19)	0.0293 (14)
N1	0.0694 (18)	0.0653 (16)	0.0443 (14)	0.0139 (14)	0.0072 (13)	0.0055 (12)
N2	0.105 (3)	0.122 (3)	0.068 (2)	0.053 (2)	0.0133 (19)	0.0078 (19)
O1	0.110 (2)	0.119 (2)	0.0542 (15)	0.0513 (17)	0.0204 (14)	0.0236 (15)
O2	0.0746 (16)	0.0726 (14)	0.0636 (14)	0.0142 (13)	0.0100 (12)	0.0111 (12)
O3	0.0666 (14)	0.0682 (13)	0.0543 (13)	0.0006 (11)	0.0105 (11)	0.0010 (11)

### Geometric parameters (Å, °)

**Table:**

C1—C2	1.381 (4)	C11—C12	1.324 (5)
C1—C6	1.386 (4)	C11—H11	0.9300
C1—C7	1.491 (4)	C12—F1	1.361 (4)
C2—C3	1.378 (4)	C12—C13	1.369 (6)
C2—H2	0.9300	C13—C14	1.383 (5)
C3—C4	1.369 (4)	C13—H13	0.9300
C3—H3	0.9300	C14—H14	0.9300
C4—C5	1.364 (4)	C15—C16	1.423 (5)
C4—Cl1	1.732 (3)	C15—C17	1.473 (4)
C5—C6	1.374 (4)	C16—N2	1.135 (4)
C5—H5	0.9300	C17—O2	1.217 (4)
C6—H6	0.9300	C17—O3	1.322 (4)
C7—O1	1.204 (4)	C18—O3	1.461 (4)
C7—N1	1.380 (4)	C18—C19	1.487 (5)
C8—C15	1.360 (4)	C18—H18A	0.9700
C8—N1	1.374 (4)	C18—H18B	0.9700
C8—C9	1.484 (4)	C19—H19A	0.9600
C9—C10	1.363 (4)	C19—H19B	0.9600
C9—C14	1.381 (5)	C19—H19C	0.9600
C10—C11	1.376 (5)	N1—H1	0.8600
C10—H10	0.9300		
			
C2—C1—C6	118.5 (3)	C11—C12—F1	120.2 (4)
C2—C1—C7	125.2 (3)	C11—C12—C13	122.1 (4)
C6—C1—C7	116.3 (3)	F1—C12—C13	117.7 (4)
C3—C2—C1	120.4 (3)	C12—C13—C14	118.1 (4)
C3—C2—H2	119.8	C12—C13—H13	121.0
C1—C2—H2	119.8	C14—C13—H13	121.0
C4—C3—C2	119.7 (3)	C9—C14—C13	120.8 (4)
C4—C3—H3	120.2	C9—C14—H14	119.6
C2—C3—H3	120.2	C13—C14—H14	119.6
C5—C4—C3	121.2 (3)	C8—C15—C16	119.2 (3)
C5—C4—Cl1	119.9 (3)	C8—C15—C17	123.8 (3)
C3—C4—Cl1	119.0 (3)	C16—C15—C17	117.0 (3)
C4—C5—C6	119.1 (3)	N2—C16—C15	178.8 (4)
C4—C5—H5	120.5	O2—C17—O3	123.6 (3)
C6—C5—H5	120.5	O2—C17—C15	124.5 (3)
C5—C6—C1	121.2 (3)	O3—C17—C15	111.9 (3)
C5—C6—H6	119.4	O3—C18—C19	107.2 (3)
C1—C6—H6	119.4	O3—C18—H18A	110.3
O1—C7—N1	121.3 (3)	C19—C18—H18A	110.3
O1—C7—C1	122.9 (3)	O3—C18—H18B	110.3
N1—C7—C1	115.7 (3)	C19—C18—H18B	110.3
C15—C8—N1	119.2 (3)	H18A—C18—H18B	108.5
C15—C8—C9	120.7 (3)	C18—C19—H19A	109.5
N1—C8—C9	120.1 (3)	C18—C19—H19B	109.5
C10—C9—C14	118.1 (3)	H19A—C19—H19B	109.5
C10—C9—C8	121.1 (3)	C18—C19—H19C	109.5
C14—C9—C8	120.6 (3)	H19A—C19—H19C	109.5
C9—C10—C11	121.1 (4)	H19B—C19—H19C	109.5
C9—C10—H10	119.4	C8—N1—C7	128.6 (3)
C11—C10—H10	119.4	C8—N1—H1	115.7
C12—C11—C10	119.7 (4)	C7—N1—H1	115.7
C12—C11—H11	120.2	C17—O3—C18	117.1 (3)
C10—C11—H11	120.2		
			
C6—C1—C2—C3	0.5 (5)	C10—C11—C12—C13	3.4 (7)
C7—C1—C2—C3	−178.5 (3)	C11—C12—C13—C14	−2.7 (7)
C1—C2—C3—C4	0.8 (5)	F1—C12—C13—C14	179.3 (4)
C2—C3—C4—C5	−1.3 (5)	C10—C9—C14—C13	1.9 (6)
C2—C3—C4—Cl1	177.9 (3)	C8—C9—C14—C13	−174.7 (4)
C3—C4—C5—C6	0.6 (5)	C12—C13—C14—C9	0.0 (7)
Cl1—C4—C5—C6	−178.6 (3)	N1—C8—C15—C16	176.6 (3)
C4—C5—C6—C1	0.7 (5)	C9—C8—C15—C16	−0.5 (5)
C2—C1—C6—C5	−1.3 (5)	N1—C8—C15—C17	−2.5 (5)
C7—C1—C6—C5	177.8 (3)	C9—C8—C15—C17	−179.7 (3)
C2—C1—C7—O1	−172.5 (3)	C8—C15—C17—O2	7.0 (5)
C6—C1—C7—O1	8.5 (5)	C16—C15—C17—O2	−172.2 (3)
C2—C1—C7—N1	8.1 (5)	C8—C15—C17—O3	−173.4 (3)
C6—C1—C7—N1	−170.9 (3)	C16—C15—C17—O3	7.4 (4)
C15—C8—C9—C10	−64.0 (4)	C15—C8—N1—C7	164.6 (3)
N1—C8—C9—C10	118.9 (4)	C9—C8—N1—C7	−18.2 (5)
C15—C8—C9—C14	112.5 (4)	O1—C7—N1—C8	0.0 (5)
N1—C8—C9—C14	−64.7 (4)	C1—C7—N1—C8	179.4 (3)
C14—C9—C10—C11	−1.2 (6)	O2—C17—O3—C18	−0.1 (4)
C8—C9—C10—C11	175.4 (3)	C15—C17—O3—C18	−179.7 (3)
C9—C10—C11—C12	−1.4 (6)	C19—C18—O3—C17	−179.3 (3)
C10—C11—C12—F1	−178.6 (4)		

### Hydrogen-bond geometry (Å, °)

**Table:**

*D*—H···*A*	*D*—H	H···*A*	*D*···*A*	*D*—H···*A*
N1—H1···O2	0.86	2.00	2.668 (3)	134
C3—H3···N2^i^	0.93	2.53	3.314 (5)	143
C6—H6···O1^ii^	0.93	2.41	3.170 (4)	140
C14—H14···N2^iii^	0.93	2.55	3.463 (5)	169

Symmetry codes: (i) −*x*, *y*−1/2, −*z*+1/2; (ii) −*x*+1, −*y*+1, −*z*; (iii) *x*+1, *y*, *z*.
